# Supplementary material for: Effects of climate and environmental heterogeneity on the phylogenetic structure of regional angiosperm floras worldwide
Source: Nat Commun. 2024 Feb 5;15:1079. doi: 10.1038/s41467-024-45155-9 (PMC10844608; doi:10.1038/s41467-024-45155-9)
Supplement: Supplementary file 1 — Supplementary Information [file 41467_2024_45155_MOESM1_ESM.pdf]

Supplementary Information for
Effects of climate and environmental heterogeneity on the phylogenetic structure of regional angiosperm floras worldwide

This PDF file includes:
Supplementary Figures 1 and 2
Supplementary Table 1
Supplementary Note 1

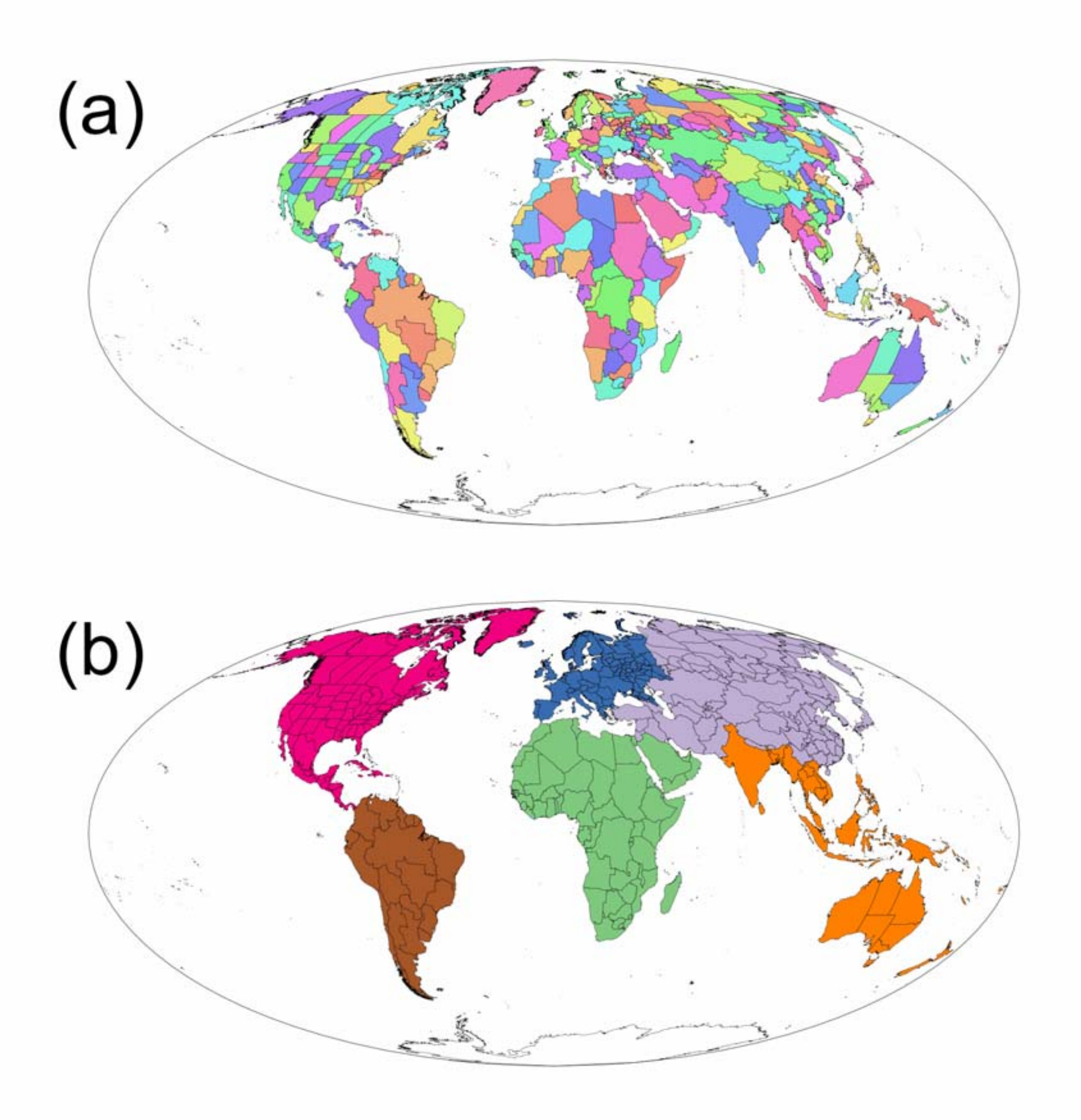

Supplementary Fig. 1. (a) Geographic regions used in this study. (b) The six geographic regions indicated by six different colors were considered as six continental regions in this study (i.e., Europe, Asia, Northern America, Africa, Australasia, and Southern America).

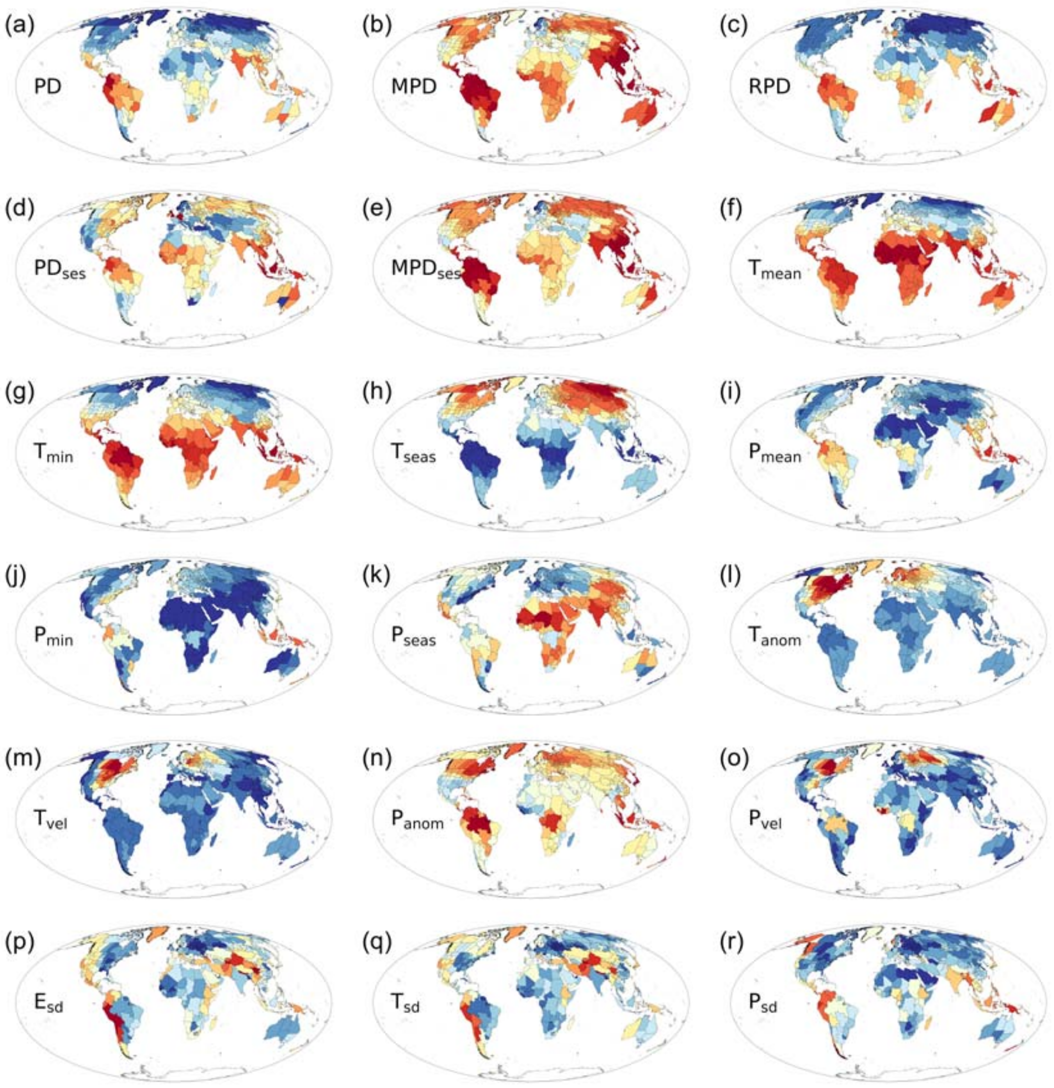

Supplementary Fig. 2. Geographic variation of each of the phylogenetic metrics and environmental variables used in this study. The color gradient from blue to red reflects values varying from small to large in each map. Full names and abbreviations of phylogenetic metrics: phylogenetic diversity (PD), mean pairwise distance (MPD), relative phylogenetic diversity (RPD), the standardized effect size of phylogenetic diversity (PD_{ses}), and the standardized effect size of mean pairwise distance (MPD_{ses}). Full names and abbreviations of environmental variables: mean annual temperature (T_{mean}), minimum temperature of the coldest month (T_{min}), temperature seasonality (T_{seas}), annual precipitation (P_{mean}), precipitation during the driest month (P_{min}), and precipitation seasonality (P_{seas}), temperature anomaly (T_{anom}), precipitation anomaly (P_{anom}), temperature velocity (T_{vel}), precipitation velocity (P_{vel}), and the standard deviations of elevation (E_{sd}), mean annual temperature (T_{sd}), and annual precipitation (P_{sd}) within regions.

Supplementary Table 1. Coefficient of determination (R^2) of regression of each of the five phylogenetic metrics against all the 13 environmental variables used in this study for the globe as a whole and for each of the six biogeographic continents.

		Biogeographic continent						
Metric	Globe	EUR	AS	NAM	AFR	AUS	SAM	Average
PD	0.539	0.682	0.769	0.821	0.340	0.583	0.896	0.682
MPD	0.269	0.704	0.658	0.730	0.697	0.814	0.874	0.746
RPD	0.643	0.620	0.829	0.788	0.445	0.639	0.888	0.702
PD_{ses}	0.486	0.561	0.671	0.788	0.640	0.647	0.914	0.704
MPD_{ses}	0.230	0.692	0.642	0.760	0.631	0.551	0.761	0.673
Average	0.433	0.652	0.714	0.777	0.551	0.647	0.867	0.701

Abbreviations of phylogenetic metrics: PD = area-corrected phylogenetic diversity, MPD = mean mean pairwise distance, RPD = relative phylogenetic diversity, PD_{ses} = standardized effect size of phylogenetic diversity, MPD_{ses} = standardized effect size of mean pairwise distance.

Abbreviations of biogeographic continents: EUR = Europe, AS = Asia, NAM = Northern America, AFR = Africa, AUS = Australasia, SAM = Southern America.

Supplementary Note 1

We included India and several countries in South-East Asia as part of Australasia in order to maximize comparability in geographic extent among continental regions (Supplementary Fig. 1). We conducted an analysis to assess whether including India and several countries in South-East as part of Asia or as part of Australasia will significantly change our result. Specifically, we removed the geographic units of the Indian subcontinents and Indochina from Australasia, and included them in Asia. We then calculated adjusted the R^2 values for the relationship between each of the five phylogenetic metrics and each of the three types of environmental variables (i.e., current climate, historical climate, and environmental heterogeneity) in each of the two continental regions. We determined the degree to which the relative importance of two types of environmental variables in each pair differs between our results reported in Figure 3 and those of the analyses outlined above. We found that the relative importance of each type of environmental variables in each pairwise comparison (e.g., current climate versus historical climate) did not change in 87% of the 30 pairwise comparisons (i.e., 5 metrics by 3 environmental types by 2 continents). Thus, whether including India and several countries in South-East as part of Asia or as part of Australasia did not make a significant difference in our results.
