# Supplementary material for: Effects of climate and environmental heterogeneity on the phylogenetic structure of regional angiosperm floras worldwide
Source: Nat Commun. 2024 Feb 5;15:1079. doi: 10.1038/s41467-024-45155-9 (PMC10844608; doi:10.1038/s41467-024-45155-9)
Supplement: Supplementary file 3 — Description of Additional Supplementary Files [file 41467_2024_45155_MOESM3_ESM.pdf]

Description of Additional Supplementary Files

File Name: Supplementary Data 1

Description: Dataset used in the study.
